# Oxidative Stress and Bronchopulmonary Dysplasia: Evidences From Microbiomics, Metabolomics, and Proteomics

**DOI:** 10.3389/fped.2019.00030

**Published:** 2019-02-13

**Authors:** Letizia Capasso, Giovanni Vento, Cristina Loddo, Chiara Tirone, Federica Iavarone, Francesco Raimondi, Carlo Dani, Vassilios Fanos

**Affiliations:** ^1^Neonatology, Section of Pediatrics, Department of Translational Sciences, University of Naples Federico II, Naples, Italy; ^2^Division of Neonatology, Department of Woman and Child Health, Pediatrics area, Fondazione Policlinico Universitario Agostino Gemelli, Università Cattolica del Sacro Cuore, Rome, Italy; ^3^Neonatal Intensive Care Unit, Neonatal Pathology and Neonatal Section, Azienda Ospedaliero-Universitaria Cagliari and University of Cagliari, Cagliari, Italy; ^4^Institute of Biochemistry, Università Cattolica del Sacro Cuore, Rome, Italy; ^5^Neonatology, University Hospital Careggi, Firenze, Italy

**Keywords:** bronchopulmonary dysplasia, oxidative stress, newborn, preterm, microbiomics, metabolomics, proteomics

## Abstract

Bronchopulmonary dysplasia is a major issue affecting morbidity and mortality of surviving premature babies. Preterm newborns are particularly susceptible to oxidative stress and infants with bronchopulmonary dysplasia have a typical oxidation pattern in the early stages of this disease, suggesting the important role of oxidative stress in its pathogenesis. Bronchopulmonary dysplasia is a complex disease where knowledge advances as new investigative tools become available. The explosion of the “omics” disciplines has recently affected BPD research. This review focuses on the new evidence coming from microbiomics, metabolomics and proteomics in relation to oxidative stress and pathogenesis of bronchopulmonary dysplasia. Since the pathogenesis is not yet completely understood, information gained in this regard would be important for planning an efficacious prevention and treatment strategy for the future.

## Oxidative Stress and Bronchopulmonary Dysplasia: an Overview

Preterm newborns are particularly susceptible to oxidative stress (OS), as a consequence of different factors, such as exposure to high levels of oxygen, inflammation and inadequate defenses to oxidative injury (1). In 1998, Saugstad coined the term “oxygen radical disease of Neonatology” to indicate several pathologic conditions of neonate where OS plays a crucial role, including bronchopulmonary dysplasia (BPD), retinopathy of prematurity (ROP), periventricular leukomalacia and intraventricular hemorrhage (IVH) (2, 3). Despite the improvements in the management of BPD in Neonatal Intensive Care Units, this illness is still considered the most common pulmonary disease in infants born preterm, especially in those weighting < 1,500 g at birth (4, 5). Moreover, BPD represents the most important respiratory long-term issue affecting the respiratory function of surviving premature infant along entire life.

The first report by Northway in 1967 focused on the inflammation in the lungs of neonates with prolonged oxygen exposure. Since then, the pathology of BPD has completely changed. A “new BPD” has been characterized with less in number and larger alveoli and abnormal lung vascular remodeling. The “new BPD” affects mostly very preterm infants with an incidence around 21–47% in the babies weighing between 501 and 1,500 g at birth (6). These infants are born with lungs in the saccular stage when alveoli are just beginning to differentiate; at this early stage of lung development both prenatal determinants (e.g., maternal chorioamnionitis) and neonatal factors such as resuscitation, oxygen exposure, infection, mechanical ventilation and patent duct arteriosus, interfere with alveolar and vascular lung formation leading to a general developmental arrest. Inflammation is the common denominator of these prenatal and neonatal interferences and the production of free oxygen radicals has a predominant role (1, 6–9).

OS results each time free oxygen radicals production exceeds the capability of antioxidant enzymes to neutralize them (8); this condition is very common in premature babies because of the immaturity of antioxidant system typical of this early age of life. Lung inflammation develops when pro-inflammatory cytokines (tumor necrosis factor α, interleukin-1 or IL-1, IL-6, IL-8) are released in response to prenatal and neonatal triggering factors (chorioamnionitis, infection, mechanical ventilation etc.). These mediators recruit and activate granulocytes to release free oxygen radicals causing endothelial peroxidation, increased vascular permeability, interstitial, alveolar and airway oedema, especially in presence of high oxygen concentration (8). Infants with BPD have an oxidation pattern that is different from those who do not develop BPD. Several authors found high levels of OS markers, such as lipid or protein oxidation products, in the early stages of this disease, suggesting the important role of OS in its pathogenesis. These markers have been detected in different body fluids few hours or in the first weeks after birth in newborns that subsequently developed BPD (10–17). This statement implies that OS may start in the antenatal period, and every antioxidant therapy given after birth might not be sufficient to prevent this disease (18).

Also, the OS mediates the intracellular modification of target genes expression that regulate lung development with mechanisms not yet completely understood (9). Yet, a comprehensive understanding of BPD pathophysiology is crucial to design both preventive and therapeutic strategies.

An interesting recent study “*in vivo*” in a cohort of 451 preterm infants addresses the association of a polymorphism related to the NADPH family with oxidative stress-related complications of prematurity as BPD, RDS and ROP. This study points out that genetic polymorphism may cause variable clinical response to oxidative stress induced damage in preterm infants (19) and explains that each preterm neonate has a different susceptibility to OS related diseases.

In recent years, a fundamental role in lung development has been attributed to lipofibroblasts, differentiated lung mesenchymal cells containing lipid droplets. These cells are responsible for alveolar growth, differentiation, homeostasis, surfactant production and repair. Experimental evidences shows that hyperoxia determines the differentiation of lipofibroblasts into myofibroblasts. Since the latter are unable to guarantee normal pulmonary epithelial cell growth and differentiation, a general arrest of alveolarization typical of BPD results (20). Interestingly, the nuclear transcription factor PPARy (peroxisome proliferator-activated receptor y) which has an important role in alveolar homeostasis and injury repair, seems to be related to myofibroblasts differentiation caused by oxidative stress. Curcumin, a known PPARy agonist, has been shown to inhibit myofibroblasts differentiation involved in BPD pathogenesis in an animal model of hyperoxia induced lung injury; in a neonatal rat model, curcumin enhanced lung maturation and blocked hyperoxia induced lung injury (9).

Beta-catenin is another target of oxidative stress recently studied, that may be relevant for a therapeutic approach to BPD. Beta-catenin is a component of the cytoskeleton and plays a role in regulating transcription of genes responsible for cell proliferation, differentiation and apoptosis (21).

Beta-catenin activity has a peak during the canalicular stage and decreases normally in the saccular stage. Examination of infants who died with BPD showed the persistence of β-catenin at term that should not be found in normally developed lungs. Hyperoxia has been showed to increase β-catenin and target gene expression in the lungs of neonatal rats; inhibition of β-catenin target gene expression prevents hyperoxia-induced arrest of alveolarization. Specifically, connective tissue growth factor (CTGF) and fibronectin are β-catenin target genes that play an important role in extracellular matrix deposition and in vascular remodeling associated to new BPD as a consequence of different factors, such as exposure to high oxygen concentration. Treatment with ICG001 (an inhibitor of β-catenin) significantly decreased expression of fibronectin and CTGF under hyperoxia in rat pups and reduced alveolar impairment associated to hyperoxia (21–23).

Hyperoxia has indeed multiple impacts on surfactant too. It can increase surfactant proteins SP-A, SP-B, and SP-C, but at the same time hyperoxia causes loss of the immune defense and surfactant powers of the protein SP-A and may have an impact on the enzyme glycerol-3 phosphate acyltransferase, thus reducing the production of surfactant phospholipids (10) and potentially prolonging need of ventilator support and the risk to develop BPD.

**Figure 1:**
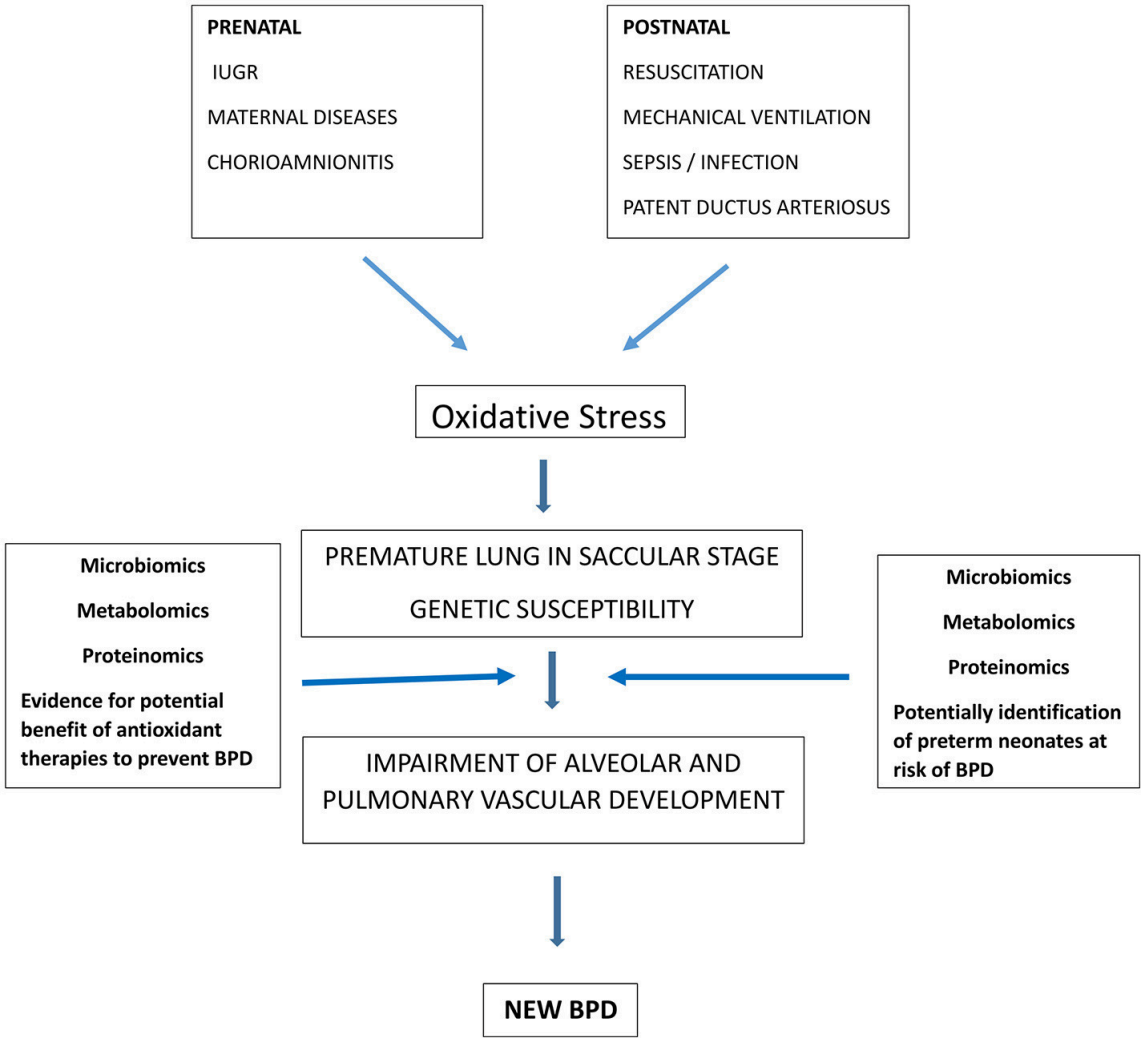
Role of oxidative stress in BPD pathogenesis and contribution of “omic sciences” to identify preterm infants at risk of BPD and to demonstrate the rationale of therapies to reduce oxidative stress and potentially prevent BPD.

BPD is a complex disease where knowledge advances as new investigative tools become available. The explosion of the “omics” disciplines has recently affected BPD research (Figure 1). The following paragraphs focus on the new evidence coming from microbiomics, metabolomics and proteomics.

## Microbiomics and BPD

Microbiomics is a field of research in which all the microorganisms of a given environment, called microbiome, are analyzed to study the potential role that such microorganisms have in diseases. An emerging field of interest is the lung microbiome and its correlation with the pathogenesis of BPD. It has been reported that there are 10–100 bacterial cells per 1,000 human cells in the upper and lower airways (24). The lung microbiome changes during the first weeks of postnatal life. In particular, there are several factors related to preterm birth that can alter its development (25). Chorioamnionitis or a transplacental infection can activate an inflammatory state that acts as a trigger for the development of BPD (26). Furthermore, the use of antibiotics both *in utero* and after birth has been associated with an increased incidence of BPD (27, 28). Other factors included are the use of ventilatory assistance devices, enteral nutrition, the occurrence of sepsis and the concomitant development of the intestinal microbiome (25). There is indeed an association (gut-lung axis) between the development of gut and lung microbiome (29). Intestinal dysbiosis has been counted among the causes of necrotizing enterocolitis, and if this process of dysbiosis occurs also at pulmonary level, it could trigger the inflammatory process underlying BPD. This could be explained by the presence of commensal bacteria in the lungs that are involved in the formation of the immune system; if such commensal bacteria are eliminated or damaged by the factors reported above, an abnormal inflammatory response responsible for the pathogenesis of BPD may take place (30).

Wagner et al demonstrated that there is a higher microbiome turnover with age in infants with a more severe BPD. In particular, the concentration of Staphylococcus spp. is lower in the first days of life, while there is an higher concentration of *Ureaplasma spp*. This suggests a possible role of microbiome in the delineation of BPD severity (31). Similarly, Imamura et al detected a higher presence of *Corynebacterium spp*. in infants with severe BPD, especially in those treated with longer invasive ventilation (32). Several authors tried to define lung microbiome in preterm infants using endotracheal aspirates (ETA) samples (33). Their findings indicate that lungs of preterm newborns are already colonized at birth with species as *Acinetobacter spp*. (34); therefore placenta and amniotic fluid are not sterile in such babies and contribute to the colonization of fetal tissues already *in utero* (24, 35).

Payne et al.(36) presented an observational study about 55 preterm infants born weighing < 1,300 grams in which ETA and nasogastric aspirates (NGA) were examined with a combined use of denaturing gradient gel electrophoresis (DGGE) and species-specific PCR. DGGE showed a wide range of bacterial species in 59% of NGA and ETA samples, many of them associated with preterm labor. Species-specific PCR found *Mycoplasma hominis* in 25% of NGA and 11% of ETA samples. With regard to patients who survived up to 36 weeks, the authors didn't find an association between *Ureaplasma spp*. in NGA and BPD, which can be related to sampling errors, even if these species are highly detected in ETA samples at 24 h of age (36).

About the role of the lung microbiome in the maturation of the immune system (37), this may act through the production of metabolites, as tryptophan catabolites, which are agonist of the aryl hydrocarbon receptor (AhR) that regulates the production of antioxidant enzymes (38, 39).

In humans, AhR is highly expressed in the lungs (40) and it exerts its anti-inflammatory effects in part by positively regulating the expression of nuclear RelB (41–44), which in turn inhibits inflammatory processes by modulating the expression of several chemokines and cytokines (45–49).

Therefore, a dysfunction in AhR can result in an increase in OS injury (50). In particular, Zhang et al showed that AhR-deficient fetal human pulmonary microvascular endothelial cells (HPMEC) are associated with an increased production of hyperoxia-induced reactive oxygen species (ROS), a heightened inflammatory state, a decrease in antioxidant enzimes and in ReIB activation compared to AhR-sufficient HPMEC (50).

Interestingly, *Lactobacilli* have the ability to metabolize triptophan into AhR agonists, suggesting an important modulatory role of these bacteria on the OS (51, 52). Segal et al revealed in their study the role of the macrolide azithromycin in increasing levels of tryptophan catabolites in ETAs of emphysematous lungs, thus reducing the production of proinflammatory cytokines (51). Moreover, a cohort study made by Lal et al. found that the number of *Lactobacilli* in the lung microbiome of preterms who developed BPD was significantly lower than infants who did not develop BPD, and this low concentration was evident in preterm newborns whose mother suffered from chorioamnionitis (53). More recently, the same authors highlighted the relationship between microbiomics and metabolomics by analyzing the metagenome of the microbiome and the metabolome at birth in the lungs of extremely low birth weight (ELBW) infants. They found an increased number of metabolites involved in sexual hormones synthesis and in fatty acid activation in the airway metabolome of BPD-predisposed infants, compared to BPD-resistant infants. The authors concluded that the lung microbiome of ELBW infants affect metabolome, and that sexual dimorphism could alter the risk of BPD (54).

Moreover, a study in mice reported that the injection of *Lactobacilli* into the lungs could improve alveolar development (55). All of these findings indicate a potential role of the microbiome in reducing the OS correlated to BPD (in fact, the administration of *Lactobacilli* or the use of azithromycin could be potential tools in the prevention or in the treatment of BPD). This can be explained by their capacity in reducing macrophage production of proinflammatory citokines, which are responsible to amplify OS (56). In the same direction, a recent study on omeprazole demonstrated that this pump inhibitor can induce Cytochrome P4501A1 (CYP1A1) that has protective properties against hyperoxia, possibly through the activation of the AhR signaling.

Omeprazole is not considered a typical AhR-ligand (57). It is assumed that it may reduce the interaction forces which hold AhR complex in a silencing state. This action could turn AhR into a DNA binding form, which if accumulated in the nucleus, could activate the transcription of CYP1A1 (58).

Therefore, omeprazole could be another potential way to prevent BPD in the prenatal and postnatal period (59).

## Metabolomics and BPD

Metabolomics is defined as “the quantitative measure of the multiparametric dynamic metabolic response of the living systems to physiopathological stimuli or genetics modifications” (60). This “omics” science studies all the final products of biochemical reactions, endogenous and exogenous metabolites, occurring in body tissues and fluids, in order to define the biochemical phenotype of an individual, as a “photograph” of the status of an organism in a specific time of his life (61–63). This aspect can be applied to better understand the predictability, the diagnosis and the treatment of several diseases, for instance BPD, for which early identification, as previously suggested, could be essential to reduce its severity and complications (64–67). Metabolomics uses two different analytical techniques, as Proton Nuclear Magnetic Resonance (1 H-NMR) Spectroscopy and Liquid Chromatography or Gas Chromatography coupled to Mass Spectrometry (LC/GC-MS), for its purposes. The process develops in two phases; first is the recognition of metabolites on the basis of their characteristic structure, followed by a multivariate statistical analysis (68). Metabolomics can be divided in two different approaches, called untargeted and targeted metabolomics. Untargeted metabolomics is a comprehensive analysis of all measurable analytes in a sample, including chemical unknowns, while targeted metabolomics is the measurement of a defined set of metabolites in a biological sample. Both the approaches have advantages and disadvantages; untargeted metabolomics takes time to process all the data and it finds difficulties in identifying and defining small molecules, in addition to the fact that it can be subject to bias, features that are reduced through the use of targeted metabolomics (69).

In 2014 V. Fanos and colleagues published the first study using this two-step process to compare urine samples collected at birth of neonates < 30 weeks who later developed BPD with a control healthy group. In the BPD group, they detected some metabolites absent in the control group, as lactate, taurine, and Trimethylamine N-oxide (TMAO), with an increased myoinositol and a decreased gluconate (69). Similar results were subsequently reported in 2016 by Baraldi et al. who examined 32 amniotic fluid samples using the same tools (70). Considering the analyzed metabolites, myinositol appears to be involved in lung maturation during antenatal life (71). The increase of the urinary lactate, on the other hand, seems to be related to the activation of anaerobic glycolysis in response to hypoxia. With regard to taurine and TMAO, these two metabolites are involved in osmoregulation, membrane stabilization and in renal cell cycle. Moreover, taurine plays other important roles in calcium homeostasis, in detoxification and in nerve cell activity. TMAO is also known as a marker of renal medullary damage (72, 73) and of papillar dysfunction (74). The authors concluded that BPD could be the sum of a dysfunctional development; consequently, it can be considered a congenital disease (75). A recent study published by the same group (76) examined urine samples at 7 days of life of 18 preterm newborns; seven of them subsequently developed BPD. The metabolites that differ in the neonates who developed BPD compared who did not, were represented by alanine, betaine, trimethylamine-N-oxide, lactate, and glycine. What is interesting is that glycine levels in BPD group were lower than in controls. Glycine is involved in glutathione synthesis, which has an important antioxidant role (77). It can also inhibit nuclear factor-kappa (NF-kB) activation and it can avoid the expression of inducible nitric oxide synthetase (iNOS) (78). Therefore, reduced levels of glycine and lactate in the BPD group could be representative of a situation of OS.

Therefore, BPD can be considered the result of a series of prenatal developmental deregulations, supporting the idea that part of the injury starts before birth (24). Consequentely, early detection of such mechanism may help to develop a targeted approach for increasing the chances of recovery of these patients. In this respect, metabolomics could be a useful tool to detect a metabolic “fingerprint” able to identify, during the intrauterine life or at birth, which newborns will later develop BPD. This could help physicians to implement its management, especially avoiding oxidative injuries as early as possible. Further studies are needed to better understand the correlation between the above findings and BPD, especially in order to find a list of the key metabolites diagnostic for this disease.

## Proteomics, Transcriptomics and BPD

Proteomics is the large-scale analysis of the proteome, the entire set of proteins produced or modified by an organism in analogy with genomics, the study of the genome. One of the positive implication of proteomics is to identify proteins implicated in diseases and offer targets to researchers to develop potential preventive strategy and treatments. Only 4% of proteomic studies aimed at lung injury and disease have focused on pediatric lung disease (79). As BPD is a multifactorial disease and involves different molecular pathways, the proteomic analysis is very important because it may help to study the networks of proteins implicated in its pathogenesis and their interactions.

Actually, no molecules are able to definitely predict the predisposition to develop BPD before its clinical onset. Several studies underlined an imbalance between proteases and anti-proteases playing a central role in the pathogenesis of BPD. An interesting study in preterm infants has been conducted by Magagnotti et al. aimed to describe the networks of proteins involved in the disease and the modulation of protein function (80). The authors showed a proteomic analysis of ETAfrom infants with BPD and controls, using mass spectroscopy. The results were further validated by western blotting. The authors found a clear differential expression in the proteome of the 23–25 weeks gestational age (GA) group respect to the 26–29 weeks GA group. Surfactant protein-A2, annexin-3, calcium and integrin binding protein-1, leukocyte elastase inhibitor, chloride intracellular channel protein 1 and calcyphosine were differentially expressed in severe BPD patients with lower GA (23–25 weeks gestation). In relation to the OS, leukocyte elastase inhibitor was downregulated in severe BPD patients. A major limitation of this study was the small sample size.

More recently, Shrestha et al. (81), combined transcriptomic and proteomic analysis to further elucidate the molecular mechanisms underlying BPD. They conducted a study using neonatal C57BL6J wild-type mice, with the aim to test the hypothesis that hyperoxia exposure during the saccular and alveolar development phase alters the level of genes and proteins necessary for optimal lung development and repair. The authors performed a correlation analysis between the proteinomic and transcriptomic data and the results showed that hyperoxia exposure dysregulated the expression of 344 genes and 21 proteins. Interestingly, hyperoxia downregulated genes were involved in maturation of lung tissues and neuronal tissue too. Oxidoreductase activity, apoptosis, plasma membrane integrity, organ development, angiogenesis, cell proliferation and mitophagy have been identified as the predominant processes affected by hyperoxia.

A proteolytic enzyme having a potential role in the pathogenesis of BPD is alpha-1 antitrypsin (AAT). AAT is a well characterized anti-proteases inhibitor of Neutrophil Elastase (NE). AAT proteinases degradation and inactivation lead to increased NE activity. AAT has significant anti-inflammatory properties leading also to its potential therapeutic use in several important diseases (82). Recently, it has been demonstrated the presence of sufficient AAT elastase inhibitory activity in the airways of a new BPD baboon model but not in the severe BPD one (83). The same authors showed an increase of AAT elastase inhibitory activity in “severe BPD” baboons following the treatment with a catalytic antioxidant, suggesting that prevention of the oxidative inactivation of AAT may be one of the mechanisms by which antioxidant therapy improves the pulmonary outcomes in animal models of “severe BPD.”

Taggart C. et al. (84) showed as the inactivation of AAT by oxidation of either methionine 351 or 358 provides a mechanism for activity regulation at sites of inflammation. On the basis of these studies, it could be hypothesized that neonates who will develop a severe new BPD form, could benefit from an increase of the AAT antiprotease activity by areosolic or endovenous administration or by treatment with a catalytic antioxidant. In neonates with respiratory distress syndrome treated with surfactant, the intravenous administration of AAT has been tested (85), but a reduction in the incidence of pulmonary hemorrhage rather than BPD was obtained. Improving research in this field could achieve a strategy to prevent BPD.

## Potential Therapies for OS Predisposing to BPD

Studies are in progress on curcumin (PPARy agonist), inhibitor of β-catenin, treatment with ICG001, lactobacilli and azithromicine as reported above.

A well-known drug, caffeine, demonstrated a significant clinical role in reducing BPD rates when used as an early treatment from the first 3 days of life. As a possible basic science explanation of this clinical finding, a recent animal study showed that caffeine ameliorates hyperoxia induced lung injury in rat pups trough attenuation of endoplasmic reticulum stress. Caffeine also attenuated the hyperoxia induced activation of cyclooxygenase-2 and markers of apoptosis as caspase-12 and BCl2/Bax that play a central role in endoplasmic reticulum stress mediated apoptosis (86).

Also, the phosphodiesterase inhibitor pentoxifylline, has been shown in an animal model of hyperoxia-induced lung -injury to increase lung antioxidant enzymes such as superoxide dismutase, catalase and glutathione peroxidase (87). This ability to increase lung antioxidant enzymes may be particularly relevant for premature babies that are prone to oxidative stress damage because of the low activity of antioxidant system in early stage of life. The development of drugs with antioxidant effect may offer in the future an important preventive strategy for BPD.

Another future strategy to prevent/treat BPD is the use of stem cell therapy based on stem cell potential in injury repair. In an animal model of hyperoxia-induced lung injury, a protective role of stem cells has been shown attributable to capacity of such cells to decrease inflammation, repair tissue and reduce apoptosis (88). As reported by Lesage and Th'ebaud recently (89), mesenchymal stromal cells (MSCs) present in perinatal tissue (placenta, cord, and cord blood) and administrated in the airway or intravenously, prevents oxygen-induced lung injury in neonatal rats and mice. Human cord blood and cord tissue-derived MSCs injected intratracheally or intravenously were also capable of preventing oxygen-induced impairment in alveolar and lung vascular growth, as well as preserving lung. MSCs seem to act by means of transferring cellular organelles such as mitochondria and secretion of bioactive molecules that modulate the repair process in the injured cells and promote lung growth. MSCs also release anti-inflammatory factors such as tumor necrosis factor-stimulated gene-6 and factors decreasing OS such as stanniocalcin-1. The therapeutic effects of MSCs on BPD seem related indeed to their paracrine effects on lung tissue, secretome, and represents a promising therapeutic approach that needs to be further explored in the future to treat BPD.

## Conclusion

BPD is the most common and serious chronic lung disease of premature infants that changed during the decades in clinical, anatomical and physiophatological features. To date, recent studies demonstrated the pivotal role of OS in influencing gene expression responsible for arrest of alveolarization and abnormal lung vascular development typical of the “new BPD.”

A definitive therapy for BPD has not been accomplished, yet. Understanding the molecular mechanisms that drive the premature lungs to the “new BPD” is the only way to achieve therapies based on a pathogenic approach, possibly to reach a definitive therapy.

Recent studies showed that some molecular mechanisms begin very early during fetal life and at birth and preterm babies have genetically determined different susceptibility to respond to OS. Microbiomics shows that factors interfering with lung microbiome in preterm neonates may trigger inflammatory process and the OS injury leading to BPD; indeed, the use of lactobacilli, azithromycin and omeprazole are drugs potentially protective against OS injury in preterm neonates. Metabolomics shows that preterm neonates subsequently developing BPD have a different metabolic response since birth compared with those not developing BPD such us reduction of glycine and increase of lactate that are related to OS. These findings support the idea that part of the oxidative injury starts before birth for neonates who will develop BPD. Metabolomics and microbiomics in the future could be used to detect a “fingerprint” able to identify at birth preterm neonates at risk of developing BPD. Early detection of such neonates may individualize neonatal management to prevent and treat OS. Proteomics shows that enzymes protecting against OS are down regulated in BPD infants, thus antioxidant therapy may be a potential tool to treat them.

In conclusion, with the help of new techniques as metabolomics, microbiomics and proteomics we may be able in the future to screen at birth the preterm new-borns more susceptible to BPD and potentially treat them in an individualized fashion with hopefully promising new therapy to prevent or attenuate BPD.

## Author Contributions

LC, GV, FR, CD, and VF conceived the manuscript and made revisions. LC and FR wrote the final version of the manuscript. GV, CT, and FI wrote the paragraph on Proteomics and BPD. VF, and CL wrote the paragraph on Microbiomics, Metabolomics and BPD. LC wrote the paragraph on Overview, Potential therapies on OS and BPD.

### Conflict of Interest Statement

The authors declare that the research was conducted in the absence of any commercial or financial relationships that could be construed as a potential conflict of interest.
